# Imaging and pathological characteristics, treatment, and prognosis of pulmonary sequestration—A retrospective study of 13 cases

**DOI:** 10.1111/crj.13672

**Published:** 2023-08-02

**Authors:** Xiangjin Liu, Rongqian Wu, Shenyu Zhu, Liang Gu, Zhixian Tang

**Affiliations:** ^1^ First Clinical Medical College The Gannan Medical University Ganzhou China; ^2^ First Clinical Medical College The Nanchang University Nanchang China; ^3^ Department of Thoracic Surgery The First Affiliated Hospital of Gannan Medical University Ganzhou China

**Keywords:** imaging characteristics, pathological characteristics, prognosis, pulmonary sequestration, treatment

## Abstract

**Objective:**

This study aimed to summarize and analyze the characteristics of pulmonary sequestration to improve our understanding of this disease.

**Methods:**

Between January 2019 and April 2023, the clinical data of 13 patients with pulmonary sequestration underwent surgical treatment at the First Affiliated Hospital of Gannan Medical University.

**Results:**

The male‐to‐female ratio was 4:9, the age was 0.5 to 60 years, and the average age was 38 ± 19 years. There were 10 and 3 cases of intralobar and extralobar pulmonary sequestration, respectively. Chest enhanced computed tomography (CT) and three‐dimensional vascular reconstruction showed that the abnormal blood vessels were derived from the descending thoracic aorta in nine cases and from other blood vessels in four cases. Three patients underwent thoracoscopic lobectomy, two underwent thoracoscopic segmentectomy, and eight underwent thoracoscopic wedge resection. All the patients successfully completed the surgery and were discharged postoperatively.

**Conclusions:**

Some patients with pulmonary sequestration exhibit no obvious symptoms. Patients with clinical symptoms are easily confused for pneumonia, bronchial cysts, lung abscesses, and lung tumors; therefore, patients with pulmonary sequestration are prone to missed diagnosis and misdiagnosis. Currently, enhanced chest CT combined with three‐dimensional vascular reconstruction can accurately show the course, branches, and relationship with the mass of the feeding artery. Routine pathological examination is helpful to further clarify the diagnosis of pulmonary sequestration. Minimally invasive thoracoscopic surgery is the preferred treatment for patients with pulmonary sequestration. Surgical resection is safe and feasible, and satisfactory results are typically obtained.

## Graphical abstract

Thoracoscopic lobectomy, thoracoscopic wedge resection of the lesion, or thoracoscopic anatomical segmentectomy can be selected based on a comprehensive analysis of imaging results. Careful and reasonable treatment of the abnormal blood supply artery of the isolated lung is the key to the operation.

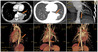

## 1 INTRODUCTION

Pulmonary sequestration was first described by Pryce DM in 1946.^1^ Pulmonary sequestration is a rare congenital pulmonary malformation, accounting for 0.15%–6.4% of all congenital pulmonary malformations.^2^ During embryonic development, owing to pulmonary artery hypoplasia, the blood supply to a part of the lung tissue is obstructed, and it is supplied by one or more abnormal arteries. These arteries are often derived from the thoracic and abdominal aortas and rarely from the main celiac, intercostal, and left gastric arteries. The veins mainly return to the pulmonary vein or systemic circulation.^3^, ^4^ The lung tissue in this area is supplied by the abnormal arteries that form a cystic mass without respiratory function. Intralobar pulmonary sequestration is defined as having a complete visceral pleura and a clear boundary separating it from the normal lung tissue; otherwise, it is called extralobar pulmonary sequestration.^5^, ^6^ The clinical manifestations of pulmonary sequestration differ. Some patients have no obvious symptoms and are diagnosed only during physical examination. Some patients had infectious manifestations, such as fever, chills, cough and expectoration, hemoptysis, and chest pain.^7^, ^8^, ^9^ The incidence of this disease is very low, and its clinical manifestations are complex and variable. Therefore, pulmonary sequestration may be easily misdiagnosed or overlooked. To analyze and improve our understanding of the imaging features, treatment methods, pathological features, and prognosis of pulmonary sequestration, we aimed to review the data of a group of patients diagnosed with pulmonary sequestration at our hospital and the literature published in recent years related to this condition.

## 2 METHODS

Between January 2019 and April 2023, 15 patients with pulmonary sequestration were admitted to the First Affiliated Hospital of Gannan Medical College, 13 of whom underwent surgical treatment and had complete clinical, imaging, and pathological data. Two patients who were clinically diagnosed with pulmonary sequestration based on their imaging results were excluded from the study because of automatic discharge, lack of surgical treatment, and lack of pathological data. Relevant data from 13 patients diagnosed with pulmonary sequestration were retrospectively collected, including sex, age, clinical symptoms, imaging examination, treatment methods, pathological features, and prognostic information. Date were analyzed using IBM SPSS Statistics version 26.

## 3 RESULTS

The clinical data of the 13 patients with pulmonary sequestration included in this study are shown in Table 1. Among the accompanying diseases diagnosed on admission in the 13 patients with pulmonary sequestration, two had hypertension, two had fatty liver, one had type 2 diabetes, one had pectus excavatum, one had pulmonary abscess, one had emphysema, and one had left renal calculi with hydronephrosis. Other patients with pulmonary sequestration did not have any accompanying diseases. Preoperatively, four patients (one each) were misdiagnosed with peripheral lung cancer in the left lower lobe, a bronchogenic cyst in the left lower lobe, a foregut cyst, and an esophageal cyst. Among the cases of intralobar pulmonary sequestration, nine were in the left lower lobe, with one in the right lower lobe. Among the cases of extralobar pulmonary sequestration, one was located in the lower lobe of the left lung, two were in the lower lobe of the right lung, and one was accompanied by a funnel chest. Imaging revealed no obvious abnormal blood supply for four patients. According to the surgical records, the abnormal blood vessels were not from the thoracic aorta but from the branches from which the arteries were not specifically described. No obvious postoperative complications occurred in any patient. All patients recovered completely, and the symptoms disappeared.

**TABLE 1:** Clinical data of 13 patients with pulmonary sequestration.

Demographic of patient	Number of patients (%)
Age at diagnosis	38 ± 19
Sex	
Female	9 (69.23%)
Male	4 (30.77%)
Symptoms	
Chest pain	3 (23.08%)
Hemoptysis	1 (7.69%)
Cough and sputum	3 (23.08%)
Asymptomatic	6 (46.15%)
Accompanying diseases	
Yes	9 (69.23%)
No	4 (30.77%)
Preoperative diagnostic	
Pulmonary sequestration	7 (53.85%)
Suspected pulmonary sequestration	2 (15.38%)
Misdiagnosed as other diseases	4 (30.77%)
Types of pulmonary sequestration	
Intralobar pulmonary sequestration	10 (76.92%)
Extralobar pulmonary sequestration	3 (23.08%)
Location of lesions detected by CT	
Left lower lobe	10 (76.92%)
Right lower lobe	3 (23.08%)
Source of blood supply	
Thoracic aorta	9 (69.23%)
Non‐thoracic aorta	4 (30.77%)
Postoperative rehabilitation	13 (100%)
Chest CT results showed	
Solid mass shadows	7 (53.85%)
Cystic shadows	3 (23.08%)
Patchy dense shadows	2 (15.38%)
Showing ground glass‐like changes	1 (7.69%)
Surgical approach	
Thoracoscopic wedge resection	8 (61.54%)
Thoracoscopic lobectomy	3 (23.08%)
Thoracoscopic segmentectomy	2 (15.38%)

**FIGURE 1:**
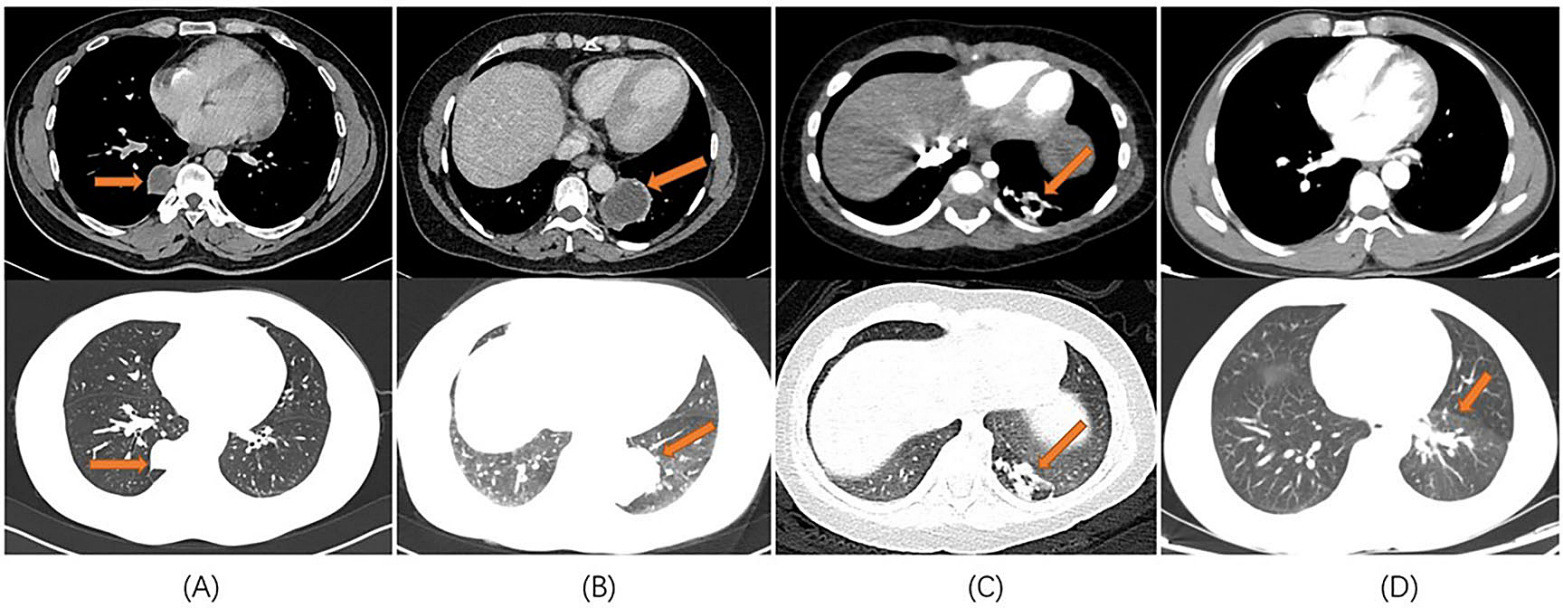
Chest CT images of patients with pulmonary sequestration (mediastinal window + pulmonary window). (A) The lumpy solid mass in the lower lobe of the right lung has uniform density and clear boundaries. (B) An irregular mixed‐density cystic shadow can be observed in the lower lobe of the left lung, with septation and scattered small patchy calcifications at the edge. (C) Patchy, dense shadows can be observed in the posterior basal segment of the left lower lobe, with unclear boundaries and reduced density of the adjacent lung parenchyma. (D) The lower lobe of the left lung shows a patchy increase in density and the boundary is slightly blurred, showing ground glass‐like changes.

**FIGURE 2:**
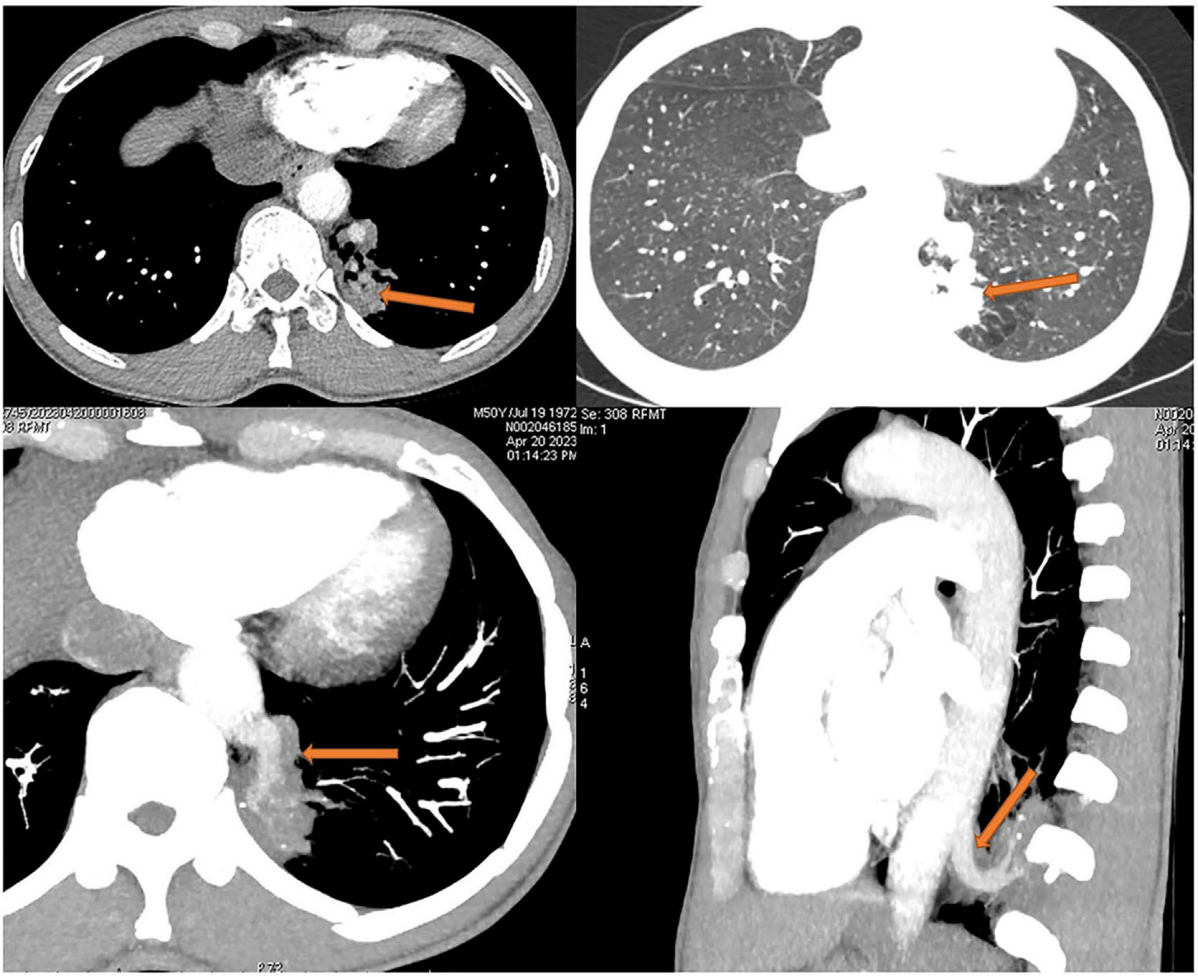
The thick branches of the thoracic aorta with a diameter of 15 mm entered the lesion. Chest enhanced CT combined with three‐dimensional vascular reconstruction shows that the posterior basal segment of the left lower lobe has patchy high‐density and unclear boundaries, and the descending thoracic aorta has a large branch with a diameter of 15 mm into the lesion.

**FIGURE 3:**
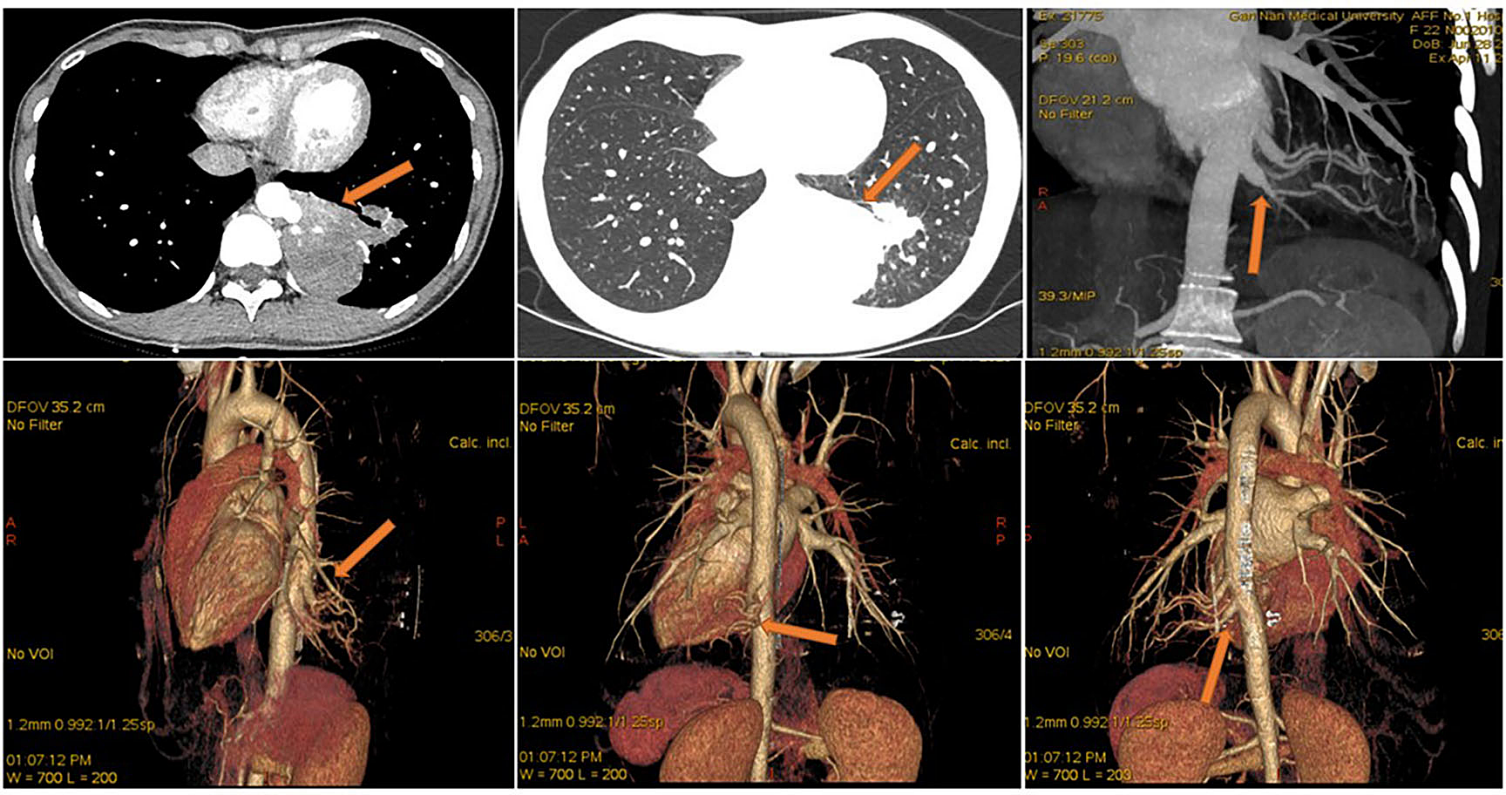
The thoracic aorta sent out three branch arteries into the lesion. Enhanced chest CT combined with three‐dimensional vascular reconstruction shows a mass‐like density shadow in the lower lobe of the left lung, uneven enhancement, and a strip shadow around it. Three branch arteries from the thoracic aorta enter the lesion.

All 13 patients underwent chest computed tomography (CT) examination (Figure 1). Chest enhanced CT combined with three‐dimensional vascular reconstruction in seven patients showed that the descending thoracic aorta had branch vessels entering the lesion site, and the imaging physician initially diagnosed it as pulmonary sequestration. Among them, six patients had one branch vessel entering the lesion, and the diameter of the thickest branch vessel was 15 mm (Figure 2). One patient had three abnormal branch arteries in the lesion (Figure 3).

**FIGURE 4:**
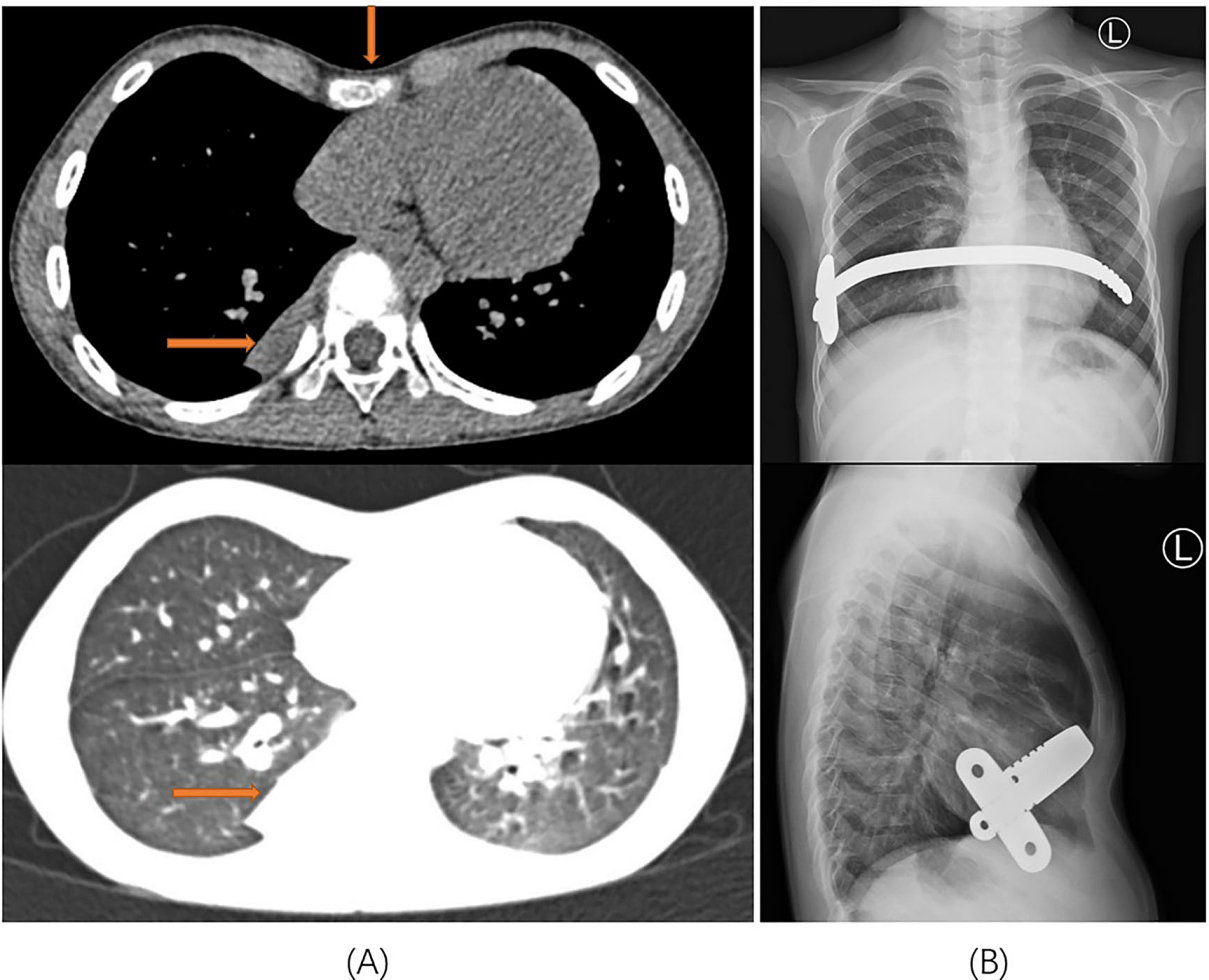
Preoperative chest CT and chest X‐ray 2 months after operation in patients with pulmonary sequestration complicated with pectus excavatum. (A) Preoperative chest CT tomography showing that the middle segment of the sternum and the corresponding bilateral costal cartilage are symmetrically depressed, with a range of approximately 13 × 14 cm, and the deepest depression was approximately 2.0 cm. A soft tissue density shadow can be observed in the lower lobe of the right lung, and pleural thickening can be observed between the two lung lobes. (B) Two months after surgery, the patient returned to the hospital for review of chest radiographs in the front and side positions. The orthopedic plate was well fixed, thoracic depression significantly improved, and the left lower lobe changed after the operation.

All 13 patients successfully underwent minimally invasive thoracoscopic surgery to remove the diseased tissue. Pleural adhesions were found in seven patients during the operation, and thoracoscopic pleural adhesion release was performed. One patient had pectus excavatum and underwent the thoracoscopic Nuss procedure after thoracoscopic wedge resection of the lesion (Figure 4). The intraoperative blood loss in 13 patients was 50–600 mL, with an average blood loss of 132.31 ± 161.97 mL. The operation time of all patients was 55–199 min, and the average operation time was 105 ± 45.78 min. A thoracic drainage tube was placed postoperatively in all the 13 patients. The time of indwelling thoracic tube was 2–5 days, and the average time of indwelling thoracic tube was 3.46 ± 0.97 days. The postoperative hospital stay of all patients was 3–10 days, and the average postoperative hospital stay was 7.08 ± 2.22 days. The total hospitalization time of patients was 4–16 days, and the average total hospitalization time was 11.31 ± 3.45 days.

**FIGURE 5:**
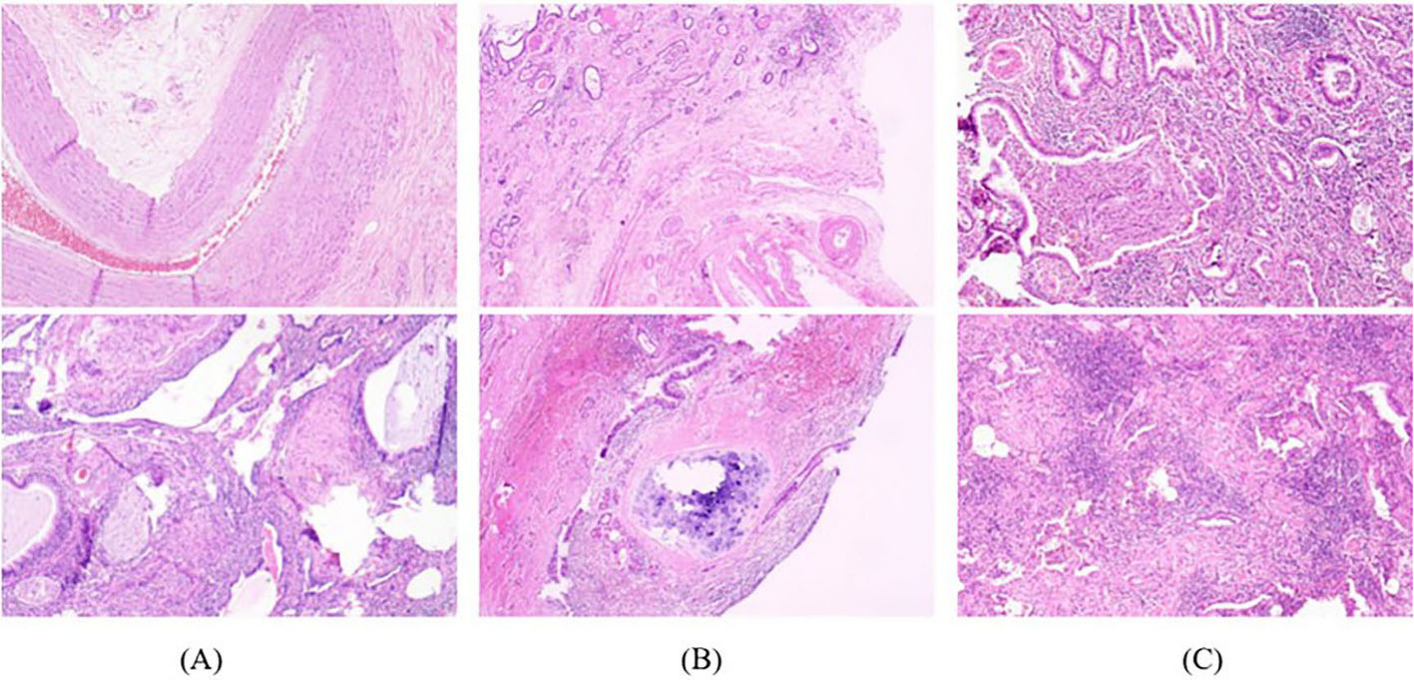
Pathological morphology of pulmonary sequestration lesions under microscope (hematoxylin and eosin stain, ×100).

Specimens from the 13 cases of surgical resection were fully drawn by more than two pathologists and evaluated under a microscope. The lesion was either cystic or solid, with an irregular honeycomb appearance, and a clear boundary from the surrounding tissue. Most cysts contained mucus‐like liquid, and a few contained thick yellow liquid or grayish‐white jelly. Under the microscope, most of the submitted specimens showed hyperplasia of the pulmonary interstitial fibrous tissue, hyaline and mucinous degeneration of the pulmonary interstitium, irregular dilatation, and hyperplasia of the bronchus, with more surrounding lymphocyte infiltration. Abnormal vascular hyperplasia with a thickened vascular wall and varying lumen size was also observed in the pulmonary interstitium (Figure 5A). Under a microscope, some areas of the cystic wall were covered with pseudostratified ciliated columnar epithelium, bronchioles proliferated, and a small amount of lung tissue was observed in the cystic wall. Cystic wall hemorrhage, fibrous tissue hyperplasia, irregular thickening of the blood vessel wall, calcium salt deposition, lymphocyte infiltration, tissue cells, and multinucleated giant cell reaction were also observed (Figure 5B). Few specimens showed purulent secretions and bacterial colonies in the lumen when observed under a microscope. Lymphocyte, plasma cell, and neutrophil infiltration, bronchiectasis, hyperplasia, interstitial fibers, and smooth muscle hyperplasia were observed in the walls and stroma in many cases. The walls of some blood vessels thickened and varied in thickness. Foam cell deposition and cholesterol crystallization were observed on the walls (Figure 5C).

## 4 DISCUSSION

Pulmonary sequestration is a rare congenital pulmonary malformation first described by Pryce DM in 1946.^1^ Its characteristic is that the lung parenchyma without respiratory function is not supplied by the pulmonary artery but by the branch blood vessels from the thoracic aorta, abdominal aorta, celiac artery, intercostal artery, left gastric artery, and other blood vessels.^10^ The pathogenesis of pulmonary sequestration is still controversial, but most scholars agree with the traction theory proposed by Pryce DM. Depending on whether the lesion has a complete pleural and normal lung tissue boundary, it can be divided into intralobar and extralobar pulmonary sequestration. The most common type is intralobar pulmonary sequestration, and extralobar pulmonary sequestration is divided into intrathoracic and intraabdominal (including diaphragmatic) types, based on the location of the isolated lung tissue.^5^, ^11^ The intralobar pulmonary sequestration lesion is located in the lung parenchyma, whereas the extralobar pulmonary sequestration lesion is covered with the pleura. Studies have shown that the proportion of intralobar‐type lesions in the total pulmonary sequestration is approximately 3/4–9/10, and the proportion of lesions in the right lower lobe is approximately two to three times lower than that in the left lower lobe. Abnormal blood supply arteries from the aorta account for approximately 95% of cases, of which abnormal arteries from the thoracic aorta account for approximately 76.55%.^12^, ^13^ The proportion of patients with intralobar pulmonary sequestration in this study was 76.92%, and the ratio of right lower lung lesions to left lower lung lesions was 3:10, which is roughly consistent with the literature. Combined with imaging and intraoperative findings, the abnormal arteries of nine patients (69.23%) in this study originated from the branches of the descending thoracic aorta, which was slightly lower than the proportion reported in the literature. Some patients with pulmonary sequestration have chest complications such as funnel chest and diaphragmatic hernia.^14^, ^15^, ^16^ In this study, one child with pulmonary sequestration had pectus excavatum. After a comprehensive evaluation, a thoracoscopic Nuss surgery was performed simultaneously, which was consistent with the literature report.

Clinical manifestations in patients with pulmonary sequestration differ. Most patients with pulmonary sequestration have infectious manifestations, such as cough and expectoration, fever, chills, hemoptysis, and chest pain. Some patients have no obvious symptoms and are diagnosed only during physical examination.^8^, ^17^, ^18^, ^19^ Studies have found that patients with intralobar pulmonary sequestration are mainly infected, whereas those with extralobar pulmonary sequestration are mostly found during physical examination.^12^ Among the 10 patients with intralobar pulmonary sequestration in this study, six were diagnosed with cough, sputum, chest pain, or hemoptysis, and four were diagnosed by physical examination. Among the three patients with extralobar pulmonary sequestration, two were identified by physical examination, and one was treated with cough and sputum as the main symptoms. The main clinical manifestations in the two types of patients with pulmonary sequestration were consistent with those reported in the literature. In this study, six patients (46.15%) with pulmonary sequestration were found on physical examination. The maximum age of the patients was 60 years, and some patients had repeated pulmonary infections that were not diagnosed, indicating that pulmonary sequestration is easily missed in clinical practice. Therefore, clinicians should consider pulmonary sequestration in patients with persistent lower lung infections.

Compared with the previous diagnosis of pulmonary sequestration by digital subtraction angiography, the current diagnosis of pulmonary sequestration is mainly through chest enhanced CT combined with three‐dimensional vascular reconstruction technology, which can accurately display the course and branch of the feeding artery and the relationship with the mass.^13^, ^20^, ^21^ Chest CT manifestations of pulmonary sequestration are mainly divided into cystic, cystic‐solid, and solid masses, among which mass‐solid masses are the most common.^22^, ^23^ In this study, seven cases (53.85%) showed a solid mass with uniform density and clear boundaries, three cases (23.08%) showed cystic shadows, two cases (15.38%) showed patchy dense shadows, and one case (7.69%) showed a patchy increased density shadow, showing ground glass‐like changes. The chest CT manifestations of patients with pulmonary sequestration in this study were mainly solid mass shadows, followed by cystic shadows, which is consistent with the literature. Among the 13 patients diagnosed with pulmonary sequestration, seven (53.85%), two (15.38%), and four (30.77%) patients were clinically diagnosed with pulmonary sequestration, suspected of having pulmonary sequestration, and misdiagnosed with other diseases, respectively, based on their imaging results. This study shows that despite the development of current imaging technology, pulmonary sequestration is still easily missed and misdiagnosed during clinical diagnosis and treatment.

For patients with pulmonary sequestration and surgical contraindications, interventional embolization of arteries with an abnormal blood supply can be considered, but its effectiveness and safety require further study.^24^, ^25^, ^26^, ^27^ With the exception of patients with pulmonary sequestration and clear surgery‐related contraindications, minimally invasive thoracoscopic surgery is the preferred treatment for pulmonary sequestration. Compared to traditional thoracotomy, minimally invasive thoracoscopic surgery has the advantages of a small incision, less intraoperative bleeding, faster postoperative recovery, and shorter postoperative hospital stay.^14^, ^28^, ^29^, ^30^ In this study, 13 patients underwent minimally invasive thoracoscopic surgery. The procedure was successfully completed. No obvious postoperative complications were observed. The patients recovered and were discharged postoperatively. This confirms that minimally invasive thoracoscopic surgery is safe and reliable. For extralobar pulmonary sequestration, only the lesion site was removed. For intralobar pulmonary sequestration, the surgical plan can be discussed based on imaging results. Either thoracoscopic lobectomy, thoracoscopic wedge resection of the lesion, or thoracoscopic anatomical segmentectomy can be selected.^20^, ^31^, ^32^ However, regardless of the type of pulmonary sequestration, the key to the operation is to correctly identify abnormal blood supply vessels and select the appropriate method to disconnect the abnormal blood vessels, thereby reducing the risk of intraoperative bleeding. Conservative treatment of pulmonary sequestration not only has a poor therapeutic effect but also increases long‐term complications and surgical risks.^33^, ^34^ Long‐term complications included hemoptysis, lung abscesses, and empyema. Severe diseases can lead to heart failure. Symptomatic pulmonary sequestration is prone to pleural inflammatory exudation, which leads to pleural adhesions and increases the risk of surgery.^35^ In contrast, early surgical intervention can not only avoid these complications and reduce the risk of surgery but also prevent the occurrence of malignant lesions.^36^, ^37^ In this study, seven patients (53.85%) were found to have pleural adhesions during the operation, so it took time to loosen the adhesions during the operation, which may lead to an increase in the probability of intraoperative bleeding and anesthesia accidents.

This study found that the pathological specimens of 13 patients with pulmonary sequestration showed pulmonary interstitial fibrous tissue hyperplasia, irregular dilatation, and hyperplasia of the bronchus; 10 patients were found to have more lymphocyte infiltration around the bronchus; and nine patients had abnormal pulmonary interstitial vascular hyperplasia, thickened hyperplastic blood vessel walls, and different lumen sizes. Among the three patients with cough and sputum as the main symptoms, two showed lymphocyte, plasma cell, and neutrophil infiltration in the pulmonary interstitium, and purulent secretions and bacterial colonies were also observed in the bronchial lumen. In one case, there was increased lymphocyte infiltration in the pulmonary interstitium and mucus plugs in the bronchus. Although the final diagnosis of pulmonary sequestration cannot be separated from the pathological results, based on the pathological reports of 13 patients with pulmonary sequestration, we found that these pathological changes were often easily confused with diseases such as bronchiectasis, chronic obstructive pneumonia, and chronic inflammation of the lungs. Therefore, it is often necessary for thoracic surgeons, radiologists, and pathologists to work collectively and have a comprehensive discussion before making the best diagnosis.

Our research had some limitations. First, only 13 patients with pulmonary sequestration were included due to the rareness of the disease, finally diagnosed by postoperative pathology. A single‐center small‐sample study requires limited clinical experience and may feature bias. Second, its retrospective nature features inevitable shortcomings. Finally, all patients underwent surgical treatment, but the surgical methods varied. Statistical analysis was impossible because of the limited number of patients undergoing different surgical methods, precluding the determination of which surgical approach was superior. Therefore, we must accumulate more cases and perform a long‐term follow‐up of postoperative patients to make specific clinical recommendations.

In conclusion, pulmonary sequestration is a rare congenital lung malformation. Because the clinical symptoms of pulmonary sequestration are atypical, its clinical diagnosis mainly depends on contrast‐enhanced chest CT and three‐dimensional vascular reconstruction findings, with the final diagnosis being confirmed in combination with pathological results. Minimally invasive thoracoscopic surgery is the preferred treatment for patients with pulmonary sequestration, for which the key is to carefully and reasonably treat the abnormal blood supply artery of the isolated lung. Surgery can usually achieve satisfactory results.

## AUTHOR CONTRIBUTIONS

Xiangjin Liu, Shenyu Zhu and Zhixian Tang performed the surgeries. Xiangjin Liu and Rongqian Wu reviewed the literature, and contributed to manuscript drafting. Xiangjin Liu and Shenyu Zhu edited the tables and figures. Liang Gu and Zhixian Tang strictly reviewed the manuscript and polished the grammar. Zhixian Tang acquired funding and critically revised the manuscript for important intellectual content. All authors approved the final version submitted and agree on its submission to this journal.

## CONFLICT OF INTEREST STATEMENT

The authors declare that the research was conducted in the absence of any commercial or financial relationships that could be construed as a potential conflict of interest.

## ETHICS STATEMENT

The studies involving human participants were reviewed and approved by Ethics Committee of The First Affiliated Hospital of Gannan Medical University. The patients/participants provided their written informed consent to participate in this study.

## Data Availability

The original contributions presented in the study are included in the article/supplementary material. Further inquiries can be directed to the corresponding author.
